# New horizons for newborn brain protection: enhancing endogenous neuroprotection

**DOI:** 10.1136/archdischild-2014-306284

**Published:** 2015-06-10

**Authors:** K Jane Hassell, Mojgan Ezzati, Daniel Alonso-Alconada, Derek J Hausenloy, Nicola J Robertson

**Affiliations:** 1 Institute for Women's Health, University College London, London, UK; 2 The Hatter Cardiovascular Institute, Institute of Cardiovascular Science, NIHR University College London Hospitals Biomedical Research Centre, University College London Hospital & Medical School, London, UK

**Keywords:** Neuroprotection, Post Conditioning, Neonatal encephalopathy, Birth asphyxia, Melatonin

## Abstract

Intrapartum-related events are the third leading cause of childhood mortality worldwide and result in one million neurodisabled survivors each year. Infants exposed to a perinatal insult typically present with neonatal encephalopathy (NE). The contribution of pure hypoxia-ischaemia (HI) to NE has been debated; over the last decade, the sensitising effect of inflammation in the aetiology of NE and neurodisability is recognised. Therapeutic hypothermia is standard care for NE in high-income countries; however, its benefit in encephalopathic babies with sepsis or in those born following chorioamnionitis is unclear. It is now recognised that the phases of brain injury extend into a tertiary phase, which lasts for weeks to years after the initial insult and opens up new possibilities for therapy.

There has been a recent focus on understanding endogenous neuroprotection and how to boost it or to supplement its effectors therapeutically once damage to the brain has occurred as in NE. In this review, we focus on strategies that can augment the body's own endogenous neuroprotection. We discuss in particular remote ischaemic postconditioning whereby endogenous brain tolerance can be activated through hypoxia/reperfusion stimuli started immediately after the index hypoxic-ischaemic insult. Therapeutic hypothermia, melatonin, erythropoietin and cannabinoids are examples of ways we can supplement the endogenous response to HI to obtain its full neuroprotective potential. Achieving the correct balance of interventions at the correct time in relation to the nature and stage of injury will be a significant challenge in the next decade.

## Background

Intrapartum-related insults at full term such as hypoxia-ischaemia (HI) are the third leading cause of global child deaths.^1^ Each year, over 0.7 million affected newborns die and 1.15 million develop acute disordered brain function known as neonatal encephalopathy (NE).^2^ NE is the second commonest preventable cause of childhood neurodisability worldwide^3^ with profound psychosocial and economic consequences for families and society. Protecting the newborn brain from injury around the time of birth is a global health priority.^4^ ^5^

## Term newborn brain injury: causes, pathogenesis and management

NE is a descriptive term for neurological dysfunction in the newborn infant, manifested by symptoms including difficulty with initiating and maintaining respiration, depression of tone and reflexes, subnormal level of consciousness, poor feeding and seizures.^6^ NE has a complex and multifactorial aetiology. For over two decades, perinatal neuroprotection research has focused on pure hypoxic-ischaemic brain injury; however, accumulating preclinical^7^ ^8^ and clinical^9^ evidences suggest the critical importance of the sensitising effect of inflammation.

**Figure 1:**
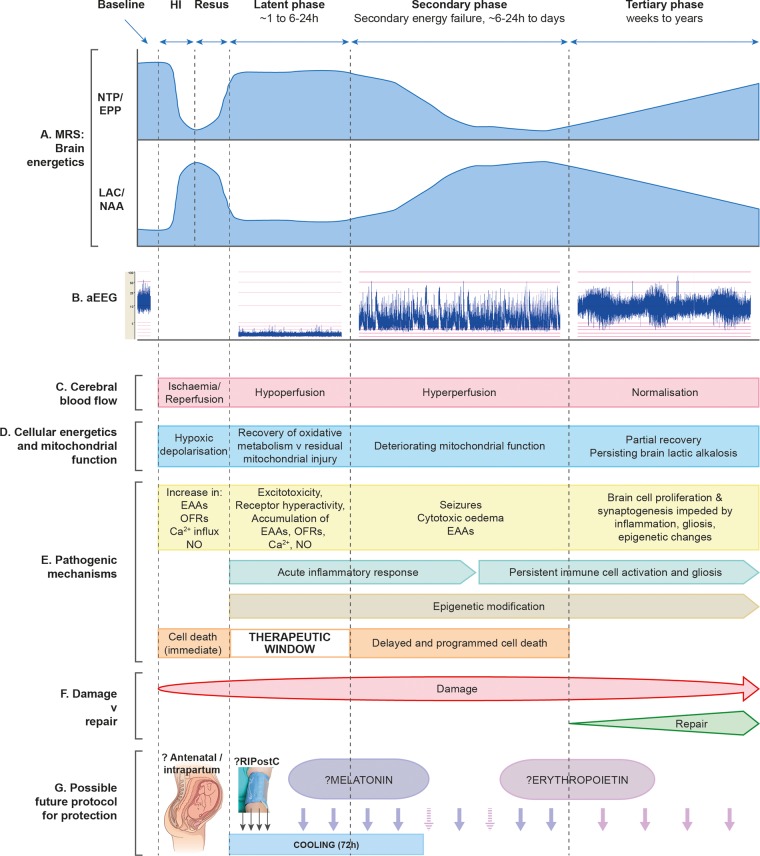
Schematic diagram illustrating the different pathological phases of cerebral injury after cerebral HI. The primary phase (acute HI), latent phase, secondary energy failure phase and tertiary brain injury phase are shown. (A) Magnetic resonance spectra showing the biphasic pattern of NTP/EPP decline and lactate/NAA increase during primary and secondary phases following HI insult. Persisting lactic alkalosis is shown in tertiary phase. (B) Amplitude-integrated EEG showing normal trace at baseline, flat tract following HI, burst-suppression pattern in latent phase, emergence of seizures in secondary phase and normalisation with sleep–wake cycling in tertiary phase. (C) Following HI, there is a period of hypoperfusion associated with hypometabolism during latent phase, followed by relative hyperperfusion in secondary phase. (D) Cellular energetics and mitochondrial function are reflected in the biphasic response shown on magnetic resonance spectroscopy (A), with a period of recovery in latent phase followed by deterioration in secondary phase. There is partial recovery in tertiary phase. (E) The most important pathogenic changes are shown for each phase (see main text for description), including generation of toxic free radical species, accumulation of EAAs, cytotoxic oedema, seizures and inflammation. Cell lysis occurs immediately following HI, while programmed cell death occurs in secondary phase; latent phase provides a therapeutic window. Persisting inflammation and epigenetic changes impede long-term repair. (F) Damage is maximal in the secondary phase, but persists into the tertiary phase as inflammation and gliosis evolve. (G) In the future, neuroprotective treatments are likely to involve a ‘cocktail’ of therapies to be administered intrapartum, in the latent phase to prevent secondary energy failure and through secondary and tertiary phases to offset evolving damage. HI, hypoxia-ischaemia; EAAs, excitatory amino acids; EPP, exchangeable phosphate pool; NAA, N-acetylaspartate; NO, nitric oxide; NTP, nucleoside triphosphate (this is mainly ATP); OFRs, oxygen free radicals; RIPostC, remote ischaemic postconditioning.

The clinical signs of NE progress after a latent period of hours to days. This timing of the evolution of clinical signs is thought to reflect brain energy levels and the cascade of neurochemical processes responsible for brain injury. These are summarised in figure 1 and described in more detail below.

### Acute HI

During the acute hypoxic-ischaemic insult, some cells undergo primary cell death, the magnitude of which will depend on the severity and duration of HI. In the absence of substrates (oxygen, glucose), the neuron's supply of high-energy metabolites such as ATP falls below a critical threshold. The Na+/K+ ATP-dependent pump begins to fail, neuronal depolarisation occurs and the synaptic cleft floods with glutamate, which activates the N-methyl-D-aspartate (NMDA) receptor. Toxic cytoplasmic Ca^2+^ concentrations arise through several mechanisms, including overactivation of glutamate receptors (NMDA, a-amino-3-hydroxy-5-methyl-4-isoxazolepropioinic acid (AMPA)), other channels and transporters, or through release from internal stores through physical damage to mitochondria and endoplasmic reticulum^10^; the increased Ca^2+^ triggers many downstream neurotoxic cascades. As well as generating an osmotic gradient that leads to oedema and lysis of cells, Ca^2+^ activates nitric oxide synthase, which in turn generates high levels of the toxic reactive oxygen species nitric oxide (NO•). At high concentrations, NO• reacts with superoxide (O•−) to produce peroxynitrite (ONOO−), which damages mitochondria via peroxidation and nitrosylation of membrane lipids. Consequently, mitochondrial dysfunction and membrane depolarisation develop with further release of O•− and decline in endogenous anti-oxidants such as glutathione. Ca^2+^ also triggers the activation of cytosolic phospholipases, which increase eicosanoid release leading to inflammation.

#### Latent phase

After reperfusion, the initial hypoxia-induced cytotoxic oedema and accumulation of excitatory amino acids partially resolve in 30–60 min, with apparent recovery of cerebral oxidative metabolism. It is thought that the neurotoxic cascade is largely inhibited during the latent phase, when there is endogenous inhibition of oxidative metabolism and increased tissue oxygenation.^11^ The ‘therapeutic window’ is believed to span this period. Much of our understanding of cerebral metabolism following HI has evolved through magnetic resonance spectroscopy (MRS) through which we have shown that latent phase duration is inversely related to insult severity.^12^ In the early recovery period (2–8 h after HI), MRS may provide an early marker of injury severity; an overshoot of phosphocreatine (PCr; donates phosphate to ADP to generate ATP) is associated with favourable outcome^13^ and raised cerebral lactate or inorganic phosphate (Pi) at 2 h is indicative of adverse outcome.^14^

### Secondary phase

Both preclinical^15^ and baby studies^16^ using phosphorus-31 (^31^P) MRS have demonstrated the deterioration in cerebral oxidative metabolism 6–24 h after HI (termed secondary energy failure) (figure 1). Despite adequate oxygenation and circulation, PCr and nucleotide triphosphate (NTP—mainly ATP) fell and Pi increased. Low cerebral PCr/Pi, NTP/total mobile phosphates,^16^ ^17^ increased brain lactate^18^ and an alkaline intracellular pH (pHi)^19^ in the first few days after birth were associated with neurodevelopmental impairment and increased mortality.

This secondary phase is marked by the onset of seizures, secondary cytotoxic oedema, accumulation of cytokines and mitochondrial failure (figure 1). Mitochondrial failure is a key step leading to delayed cell death. The degree of energy failure influences the type of neuronal death during early and delayed stages,^20^ ^21^ and the degree of trophic support influences the angiogenesis and neurogenesis during the recovery phase after HI.

### Tertiary phase

There is evidence that active pathological processes occur for weeks, months and years after a hypoxic-ischaemic insult; this has been termed tertiary brain injury.^22^ Indeed, a persisting cerebral lactic alkalosis has been observed using MRS over the first year after birth in those infants with adverse neurodevelopmental outcomes.^18^ Mechanisms of this persisting damage involve gliosis, persistent inflammatory receptor activation and epigenetic changes.

### Endogenous neuroprotection

Brain damage and lasting functional impairment after NE are the results of a balance between injurious mechanisms (cell death, persistent inflammation) and endogenous protection (acute response, recovery, repair). Optimal therapy will demand exploitation of multiple pathways that prevent brain cell death and promote repair.^23^ Much neonatal neuroprotection research has emphasised immediate cytotoxic mechanisms; however, the brain also mounts a potent, though only partially successful, defensive response against many of the deleterious secondary mechanisms of injury.^24^ Therapies to boost the endogenous neuroprotective response are particularly attractive; they are less likely to disrupt physiological neurotransmission, so may offer more effective treatments with fewer unwanted side effects.

We discuss five interventions whose actions include the augmentation of the endogenous neuroprotective response. Birth asphyxiated babies have an endogenous cooling response;^25^ therapeutic hypothermia is already the standard clinical care for babies with moderate or severe NE. Remote ischaemic postconditioning (RIPostC) is a novel therapy, which has enormous promise as an intervention that harnesses the body's neuroprotective ‘conditioning’ mechanisms. Melatonin is known for its role in entraining the circadian rhythm;^26^ however, endogenous levels of melatonin increase after HI and exogenously administered melatonin confers brain protection.^27^ Endogenous endocannabinoids and erythropoietin (Epo), likewise increased following HI, also have a role in neuroprotection. There is expanding evidence to show that Epo confers protection that extends to the tertiary phase of injury, promoting repair.

## Therapeutic hypothermia

### Background

For over 50 years, it has been known that babies with birth depression have an endogenous cooling response.^25^ We observed this phenomenon in our pilot cooling study in a low-resource setting.^28^ After two decades of laboratory studies,^29^ ^30^ clinical trials^31^ and endorsement from regulatory bodies (http://www.nice.org.uk/guidance/ipg347), therapeutic hypothermia is now standard clinical care for moderate-to-severe NE in the UK and high-income countries.^5^

### Mechanism

Pathways underpinning hypothermic neuroprotection are covered in detail in recent reviews by Wassink *et al*^32^ and Edwards *et al*^33^ In brief, these pathways include a decrease in metabolic rate with parallel decreases in O_2_ consumption and CO_2_ production, reduced loss of high-energy phosphates during HI and during secondary cerebral energy failure, reduced excitotoxicity, reduced reactive oxygen species production, protein synthesis preservation, decreased oedema, modulation of the inflammatory cascade and a change in pro-apoptotic and anti-apoptotic signalling.^34–36^

### Clinical application

In intensive care settings, clinical trials have included whole body cooling with core temperature reduced to 33.5°C for 72 h^37^ and selective head cooling with core temperatures reduced to 34.5°C.^38^ Some studies suggest less severe brain MRI findings in babies who have had whole body cooling versus selective head cooling; other studies suggest equal benefit from both cooling methods.^39^ ^40^

There is clear evidence that therapeutic hypothermia as a therapy for moderate-to-severe NE reduces adverse outcome (mortality and neurodevelopmental disability) at 18 months of age (typical relative risk 0.75, 95% CI 0.68 to 0.83)^31^; this improvement persists into childhood^41^ and there is widespread benefit to society, individuals and the economy (UK >£125 million benefit).^42^ However, therapeutic hypothermia offers only an 11% reduction in risk of death or disability, from 58% to 47%.^4^ Moreover, effective cooling treatment requires a high level of neonatal intensive care support, which is not available in many lower resource settings. There is an urgent need to develop additional simple, safe and effective neuroprotective treatment strategies.

### Caveats of hypothermia

Recently, therapeutic hypothermia has been shown to be ineffective and even harmful in the presence of infection/inflammation in adult clinical studies.^43^ In a preclinical neonatal rodent study, cooling was not neuroprotective in inflammation-sensitised HI.^44^ In a small prospective study of placental histology relative to MRI in babies, therapeutic hypothermia was less protective in babies whose placenta showed chorioamnionitis.^45^ We reported an unexpectedly high mortality in NE cases cooled to 33.5°C in a small pilot therapeutic hypothermia feasibility (not efficacy) study in sub-Saharan Africa^28^; this may have been related in part to higher rates of intercurrent infection/inflammation in affected infants.

Brain injury and hypothermia both alter immune responsiveness. Following HI, a bidirectional communication between the injured brain and the peripheral innate and adaptive immune system regulates the progression of both ischaemic pathology and tissue repair (for an in-depth review of the dualistic role of inflammation, see An *et al*,^46^). HI acutely triggers the release of cytokines and chemokines from neurons, astrocytes and microglia. These signals induce microglial activation, trigger further release of pro-inflammatory cytokines such as tumour necrosis factor α and interleukin 6 (IL-6) and recruit white blood cells (WBCs). The infiltration of macrophages is both detrimental in ischaemic injury and protective against haemorrhage. Similarly, while early elevation of circulating neutrophils after HI may augment brain injury,^47^ prolonged immunosuppression and T cell lymphopenia is associated with immune paralysis and worse outcome in animal models of stroke,^48^ traumatic brain injury^49^ and human adult stroke.^50^ Thus, inflammation following HI has both helpful and harmful effects, which may affect the response to neuroprotective treatments.

A key mechanism of action of therapeutic hypothermia is the inhibition of the pro-inflammatory cascade^51^; and hypothermia may therefore inhibit both protective and damaging responses.^52^ A recent study investigated the effect of therapeutic hypothermia on modulating the peripheral immune response over the first 72 h after birth in 65 infants with NE.^53^ Hypothermia lowered absolute neutrophil and lymphocyte counts compared with normothermic infants. In the hypothermic group, those patients who did not have a recovery of their WBC counts after rewarming had poor outcomes, whereas those who had better recovery of WBC counts had a better long-term outcome.^53^ This may indicate immune paralysis in the adverse outcome group. It is possible, therefore, that hypothermic immune suppression has a negative influence on infants with infection-sensitised brain injury. Several adult studies of hypothermia have found higher infection rates in cooled groups.^54^ This has not been shown in neonatal studies of hypothermia treatment; however, blood culture-positive neonatal sepsis rates are low (5–12%) and much larger trials would be needed to detect any increase. A better understanding is crucial for achieving optimal neuroprotection in NE.

## Remote ischaemic postconditioning

### Background

*‘*Conditioning’ describes an adaptive process of endogenous protection that occurs in all mammalian species, in which small, sublethal doses of a harmful agent protect an organism against a lethal dose of the same agent. Conditioning paradigms include toxins, substrate deprivation and infection/inflammation.^24^ One conditioning agent may also confer protection against a different insult.^55^ ^56^

Ischaemic *pre*conditioning describes brief non-lethal episodes of ischaemia that confer protection against subsequent cell-lethal ischaemia, as has been observed in clinical studies of transient ischaemic attack^57^ ^58^ and angina.^59^ It is likely that contractions during labour also represent a preconditioning stimulus. Ischaemic *post*conditioning evolved from this concept^60^ ^61^ and is defined as intermittent sublethal interruptions to blood flow *after* the cell-lethal ischaemia.^62^ Postconditioning is effective if performed on a non-vital organ, such as a limb, remote to the affected organ^63^—called *remote* ischaemic postconditioning (RIPostC). Use of a remote limb makes RIPostC a feasible clinical treatment strategy for NE.

### Mechanism

**Figure 2:**
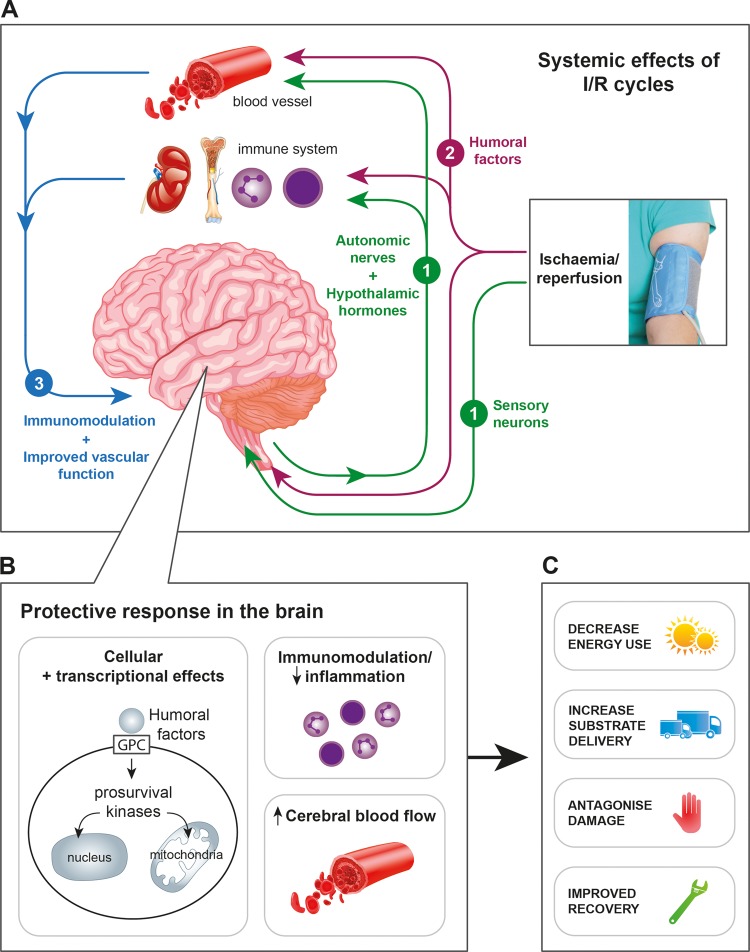
(A) The neuroprotective mechanisms of RIPostC are thought to involve three inter-related pathways induced by remote limb ischaemia. (1) The neuronal pathway involves activation of both local sensory nerves and the autonomic nervous system to mediate protective effects, including the release of humoral factors; (2) the humoral pathway involves endogenous protective factors, including locally acting autocoids and bloodborne humoral factors that travel to the brain and (3) the systemic response includes immune modulation and blood pressure regulation. (B) Within the brain, the three pathways converge to increase cerebral blood flow, ameliorate neuroinflammation and to activate cell survival mechanisms. Direct pro-survival actions within cells are mediated via G-protein-coupled (GPC) receptors and include mitochondrial protection (maintenance of potassium-sensitive ATP channel, prevention of mitochondrial permeability transition pore opening) and transcriptional regulation (both genetic and epigenetic modulation) in the nucleus. (C) Following remote ischaemic stimulus after HI, the effects of these neuroprotective mechanisms are to decrease energy consumption; to increase substrate delivery and offset cerebral secondary energy failure; to protect against cell death and to augment long-term recovery and repair. HI, hypoxia-ischaemia; I/R, ischaemia/reperfusion; RIPostC, remote ischaemic postconditioning.

RIPostC has been shown to protect the adult and neonatal brain in rodent models of stroke. The protective mechanisms of RIPostC are incompletely understood, but are thought to involve three intimately inter-related pathways initiated by the release of a number of endogenous autocoids (including adenosine, bradykinin, opioids) from the ischaemic skeletal muscle. These pathways are (i) the neuronal pathway; (ii) the humoral pathway and (iii) the systemic response (figure 2).^64^ Animal models have shown that interruption of any one of these pathways abrogates the neuroprotection conferred by RIPostC.

In brief, the neuronal pathway describes the autocoid-mediated stimulation of local afferent nerves that effect remote protection via efferent nerves, including the autonomic nervous system.^65^ ^66^ Both limb ischaemia and efferent nerve activation trigger the release of a number of bloodborne protective factors that are transported in the circulation and mediate protection in the brain—the humoral pathway.^67^ ^68^ The systemic pathway describes the impact of RIPostC throughout the body, including immune effects (such as reduced neutrophil activation) and reduced expression of apoptotic and inflammatory genes.^69^

Following remote ischaemic stimulus, these three pathways converge in the brain to increase cerebral blood flow, attenuate neuroinflammation and at a cellular level to activate pro-survival signalling cascades, including genetic and epigenetic modulation. Ultimately these processes protect mitochondrial integrity, reduce energy demands, increase cell survival and promote repair mechanisms^70–72^ (figure 2).

In neonatal^73^ and adult^74^ small animal models, RIPostC reduces infarct size in focal and global ischaemia. Moreover, these studies have shown an extended therapeutic window for the application of RIPostC following hypoxic-ischaemic brain injury^63^ and application of RIPostC up to 24 h after insult was associated with improved long-term motor outcomes.^75^

In our large animal (piglet) model of perinatal asphyxia, we found that four 10 min cycles of ischaemia/reperfusion of both lower limbs, starting immediately following resuscitation, provided protection in the white matter,^76^ with decreased cell death and inflammation. MRS data in our study showed that RIPostC mitigated the rise in white matter lactate/N-acetyl aspartate and increased whole-brain ATP, findings that predict better long-term outcome in clinical studies in human newborns.^16^ ^77^

### Hurdles to clear before clinical translation

RIPostC has been explored in clinical settings for conditions, including cardiac disease and stroke.^78^ A meta-analysis of 23 randomised clinical trials of limb conditioning in adults undergoing cardiac surgery found reduced incidence of myocardial infarction in the limb-conditioned groups, regardless of timing. A randomised control trial of RIPostC for children undergoing cardiac bypass also showed cardioprotection.^79^ In adult stroke, a recent study of 443 adults who underwent prehospital remote ischaemic perconditioning as an adjunct to thrombolysis for acute ischaemic stroke found a reduced risk of tissue infarction in the treatment group.^80^ In all studies, remote ischaemic conditioning was safe and well tolerated. However, another meta-analysis of remote ischaemic preconditioning in open cardiac surgery showed the cardioprotective effect was most marked in studies without full blinding, emphasising the need for further double-blind randomised studies.^81^

Clinical trials are needed to establish whether RIPostC is safe and protective in NE. It will be important to address the safety and reproducibility of inducing intermittent limb ischaemia, with and without concomitant cooling therapy. There remain difficult hurdles such as the dose–response of RIPostC (how many cycles and for how long achieves best protection and avoids any detrimental effects), the therapeutic time windows and the precise protective mechanisms.^82^

## Melatonin

### Background

Melatonin is a naturally occurring neuroendocrine molecule secreted in response to environmental light–dark cycles. Melatonin is both lipophilic and hydrophilic. It easily crosses biological membranes and acts via receptor-dependent and receptor-independent processes to modulate cell signalling and gene expression.^83^ ^84^ While melatonin's key and probably best-known role is to regulate the body's multifarious circadian rhythms,^26^ it influences numerous physiological functions, including growth and development, reproduction and the immune response.

Endogenous melatonin is integral to normal neurodevelopment and protects the developing brain from injury.^85–90^ Maternal melatonin levels are raised in pregnancy^91^ ^92^ and melatonin readily crosses the placenta and blood–brain barrier.^93^ ^94^ Healthy term-born neonates have relatively low pineal melatonin production, which lacks diurnal variation for the first weeks of life.^95^ ^96^ However, we observed a 6- to 15-fold increase in plasma melatonin following HI in our experimental model of perinatal HI^27^ and a similar response has also been observed in human stroke and in critically ill children,^97^ ^98^ implying a role for melatonin in an endogenous protective response.

### Mechanism

Acting via specific cell membrane and nuclear receptors, melatonin achieves powerful neuroprotective effect via anti-oxidant, anti-apoptotic and anti-inflammatory processes^85–88^ and by promoting neuronal and glial development.^99–101^ Developing brain tissue is highly susceptible to free radical damage^102–104^ and the potent free radical-scavenging properties of melatonin and its metabolites provide a fundamental neuroprotective mechanism.^105–109^ Additional indirect anti-oxidant effects of melatonin include upregulation of anti-oxidant enzymes^94^ and crucially the preservation of mitochondrial integrity.^106^ ^107^ ^109^ Numerous rodent and large animal studies have shown that melatonin reduces oxidative damage to cerebral lipids^104^ ^110–116^ and notably ameliorates secondary cerebral energy failure^27^ and apoptosis.^116–121^

Further, melatonin's wide-ranging immune-modulating properties^122^ ^123^ facilitate neuroprotection following HI.^27^ ^118^ ^121^ ^124^ Importantly, melatonin is protective in lipopolysaccharide-sensitised HI.^125^ Given the evidence outlined above indicating therapeutic cooling may lack efficacy following infection-sensitised HI,^44^ ^45^ melatonin may prove an effective immune-modulating neuroprotectant in such cases.

### Clinical use and safety

Melatonin is an extremely safe neurotherapeutic. No study of antenatal or postnatal melatonin treatment has shown any serious side effects,^126^ nor were any serious adverse events identified in 3000 children taking melatonin for up to 6 years.^127^ In small neonatal clinical studies, melatonin improved outcomes in sepsis,^128^ prematurity^129^ and perinatal asphyxia.^130^ In our large animal model of perinatal asphyxia, we showed neuroprotective efficacy conferred by melatonin-augmented cooling when compared with cooling alone.^27^ In our study, melatonin 30 mg/kg (which is 100 times the dose administered for disordered sleep in children) administered to newborn piglets immediately after HI over 6 h did not alter any physiological variable.^27^ A study of 30 term infants with NE randomised to cooling alone or cooling plus oral melatonin (five daily doses of 10 mg/kg per day made up from melatonin tablets crushed and dissolved in distilled water) suggested improved neurological outcome at 6 months in the melatonin group.^131^ However, four patients in the hypothermia group had severe encephalopathy, whereas only two in the hypothermia/melatonin group had severe encephalopathy at birth; this may lend bias to the results in this small study. Further, the blood levels of melatonin on day 5 in the cooling group were 32.1+3.5 pg/mL, while in the melatonin/cooling group were 42.7+5.1 pg/mL; preclinical data suggest that significantly higher pharmacological levels of melatonin are needed for optimal protection and work is currently underway to determine the lowest effective dose of melatonin for neuroprotection. Nevertheless, phase I and II clinical studies of melatonin-augmented hypothermia for NE are keenly awaited. In 2011, melatonin was rated by an international group of leading perinatal neuroscientists as the most promising of 13 neuroprotectants nearing clinical translation.^132^

## Cannabinoids

### Background

Endocannabinoids are emerging as a potential neurotherapeutic for NE. The endocannabinoid system is a neuromodulatory system that participates in a wide range of physiological processes in mammals.^133^ This endogenous system consists of target receptors, endogenous ligands and the enzymes responsible for endocannabinoid biosynthesis, transport and degradation.^134^ ^135^ Accumulating evidence indicates that endocannabinoids, like melatonin, are inherently involved in the normal development of the fetal central nervous system and its functions.^136–141^ Moreover, the levels of endocannabinoids, which are normally found at low concentrations in the brain, dramatically increase upon neuronal injury,^136^ ^142–144^ suggesting that endocannabinoids provide an endogenous neuroprotective system.^145^

### Mechanism

Endocannabinoids modulate the intensity and extension of neurotoxic processes^146–150^ and the inflammatory response^151–155^ and promote cell survival.^156–161^ Synthetic cannabinoid agonists have shown significant grey and white matter protection in animal studies of brain injury.^162–167^ In large animal models of perinatal asphyxia, cannabinoid WIN55212-2 administered immediately after HI protected mitochondrial injury and prevented apoptosis.^162^ ^163^ Cannabidiol given immediately after HI reduced neuronal injury, cerebral haemodynamic impairment, brain oedema and seizures and restored motor and behavioural performance in the 72 h after HI.^166^ ^167^ In rodent models of stroke, prolonged 7-day administration of cannabinoid WIN55212-2 started immediately after injury enhanced long-term neuronal and oligodendrocyte recovery and regeneration.^164^ ^165^ Cannabinoids, however, achieve neuroprotection in part through hypothermia. For example, the cannabinoid agonist HU10 induced hypothermia in an experimental stroke model and was protective, but this benefit was completely abolished by rewarming animals to the temperature of the control group.^168^

### Clinical use and safety

The main established clinical uses of cannabinoids are for chronic pain, for muscle spasms and as an appetite stimulant.^169^ ^170^ Additionally, the synthetic cannabinoid dexanabinol is in a number of phase I clinical trials for primary and secondary solid tumours (http://www.clinicaltrials.gov). A previous phase III randomised controlled trial of 861 adult patients given dexanabinol as a neuroprotectant following traumatic brain injury showed that dexanabinol was safe but not efficacious.^171^ However, we did not identify any reported clinical studies of cannabinoids for stroke or perinatal brain injury, or studies where cannabinoids were combined with therapeutic hypothermia.

Reported side effects of cannabinoids have all been mild and transient, including sedation, anxiety, dizziness and nausea.^169^ ^170^ Maas *et al*^171^ found no toxic cardiac, hepatic or renal effects in their study of dexanabinol for traumatic brain injury. However, cannabinoids have been shown to accumulate selectively in the brain and their clearance is relatively slow,^169^ thus preclinical pharmacokinetic studies would be imperative prior to clinical trials of cannabinoids for NE.

## Erythropoietin (Epo)

### Background

Epo is a pleiotropic cytokine with multiple roles in addition to that of a haemopoietic growth factor. As with melatonin and cannabinoids, the role of Epo in normal brain development and neuroprotection is becoming clear (for review see Rangarajan and Juul^172^). Epo receptors (EpoR) are located throughout the central nervous system on neurons,^173^ glia^174^ and endothelial cells;^175^ they participate in proliferation and differentiation of these cells^176^ and are upregulated following brain injury. In a similar way to the exaggerated hypoxic-ischaemic injury observed when the endogenous melatonin response is abolished in pinealectomised animals,^177^ the absence of endogenous Epo and EpoR augments ischaemic damage and impairs neuronal survival.^178^

Epo is a key component of the body's endogenous ‘conditioning’ response to injurious paradigms, including ischaemia. Hypoxic preconditioning occurs when Epo is expressed after brief hypoxia, reducing damage following a second insult.^179^ This effect can be replicated by treatment with exogenous Epo prior to HI.^180^ Preclinical and clinical studies have harnessed the conditioning and regenerative potential of Epo, which is now emerging as a promising neuroprotectant that promotes repair into the tertiary phase of NE.

### Mechanism

Hypoxia and pro-inflammatory cytokines activate hypoxia-inducible factor to induce expression of Epo and EpoR. Following brain injury, Epo is anti-apoptotic,^181^ anti-oxidative^182^ and anti-inflammatory.^183^ However, a key role for Epo is repair; Epo binding stimulates neurogenesis, oligodendrogenesis and angiogenesis, all of which are upregulated following brain injury.^184^ ^185^ Additionally, Epo increases neuronal and glial migration around the injured area via the secretion of matrix metalloproteinases.^186^ ^187^ In animal studies of term and preterm perinatal HI, Epo treatment results in reduced brain volume loss and improved cognitive and motor outcomes^188–190^ and augments the neuroprotection conferred by cooling alone.^191^

### Epo in clinical trials

Numerous animal studies of Epo for brain injuries, including stroke and perinatal HI, have shown that high-dose recombinant Epo and Epo-mimetics are safe and cross the blood–brain barrier, resulting in neuroprotection.^192^ Epo pharmacokinetics has been studied using doses from 250 U/kg to 2500 U/kg.^193^ Phase I/II studies in human preterm^193^ and term^194^ ^195^ neonates, performed to establish feasibility, safety and appropriate dosing, have not identified any of the common side effects observed in adults (polycythaemia, thrombosis, hypertension). Epo-mimetics have been developed to improve neuroprotection without stimulating erythropoesis.^196^

Follow-up of 22 infants enrolled in a phase I clinical trial of Epo-augmented hypothermia (no comparison group) for treatment of NE found no deaths and only one infant with moderate–severe disability at age 2 years.^197^ A number of larger phase II/III studies of Epo safety and efficacy in neonatal populations are underway (reviewed in Rangarajan and Juul^172^). The optimal dose and regimen for human Epo neuroprotection is still not known; however, key points have been learnt from rodent studies such as the requirement for multiple injections and late 1 week dosing for maximal protection^198^; a study of 45 term infants comparing single-dose Epo alone on day 0 with 72 h therapeutic hypothermia alone for treatment of NE found superior protection in the hypothermia group.^199^ Further studies are needed to fully understand the specific role of Epo in the tertiary phase of brain injury and repair. Thus, the combined safety and efficacy of Epo administered alongside established and novel treatments that ameliorate secondary energy failure (cooling, melatonin) must be determined as key next steps in clinical translation.

## Conclusion

Perinatal HI leading to NE sets up a cascade of processes that lead to an evolving brain injury, which includes a latent phase, secondary energy failure phase and tertiary brain injury phase. Coexisting infection/inflammation may exacerbate this injury. The brain mounts a potent, though only partially successful, defensive response against many of the deleterious secondary mechanisms of injury. Therapies that augment the endogenous neuroprotective response such as RIPostC are attractive but need further study to define optimal protocols. Part of the endogenous neuroprotective response is lowering of the core body temperature as well as increased melatonin, Epo and cannabinoid levels. Augmenting these endogenous responses have shown protection in preclinical studies and therapeutic hypothermia is now a routine therapy for moderate-to-severe NE. Clinical trials are now ongoing for Epo-augmented hypothermia. We anticipate that future newborn brain protection will comprise a tailored combination of therapies; the challenge will be to ensure the timing and dose of each neuroprotectant are appropriate for the phase of injury to ensure optimal and lasting protection.
